# Structural, mechanical and electronic properties and hardness of ionic vanadium dihydrides under pressure from first-principles computations

**DOI:** 10.1038/s41598-020-65910-4

**Published:** 2020-06-01

**Authors:** Wenjie Wang, Chuanzhao Zhang, Yuanyuan Jin, Song Li, Weibin Zhang, Panlong Kong, Chengwu Xie, Chengzhuo Du, Qian Liu, Caihong Zhang

**Affiliations:** 1 Department of Physics and Optoelectronic Engineering, Yangtze University, Jingzhou, 434023 China; 2 School of Physical Science and Technology, Key Laboratory of Advanced Technologies of Materials, Southwest Jiaotong University, Chengdu, 610031 China

**Keywords:** Electronic properties and materials, Phase transitions and critical phenomena

## Abstract

Based on a combination of the CALYPSO method for crystal structure prediction and first-principles calculations, we explore the crystal structures of VH_2_ under the pressure range of 0−300 GPa. The cubic *Fm-*3*m* phase with regular VH_8_ cubes is predicted to transform into orthorhombic *Pnma* structure with fascinating distorted VH_9_ tetrakaidecahedrons at 47.36 GPa. Both the *Fm-*3*m* phase at 0 GPa and the *Pnma* phase at 100 GPa are mechanically and dynamically stable, as verified with the calculations of elastic constants and phonon dispersions, respectively. Moreover, the calculated electronic band structure and density of states indicate both stable phases are metallic. Remarkably, the analyses of the Poisson’s ratio, electron localization function (ELF) and Bader charge substantiate that both stable phases are ionic crystals on account of effective charges transferring from V atom to H. On the basis of the microscopic hardness model, the *Fm*-3*m* and *Pnma* crystals of VH_2_ are potentially incompressible and hard materials with the hardness values of 17.83 and 17.68 GPa, respectively.

## Introduction

High pressure is an interesting external condition, which can reduce interatomic separation and alter the bond angles and generate the powerful influences on the physical and chemical properties and further arouse unusual stoichiometries and crystal structures in numerous materials. Hydrides have been widely progressed under high pressure due to potential high-temperature superconductors and hydrogen storage material^1–11^.

Recently, the investigations on the transition metal hydrides under high pressure have also been conducted in views of two factors as follows. On the one hand, for the late transition metals, their electronegativity values (e.g., 2.2 for Ru, Pd, Os, and Ir; 2.28 for Pt; 2.54 for Au) are higher and comparable than that of hydrogen (2.2) and thus making the chemical reaction between them unfavorable and resulting in the extreme absence of the late transition metal hydrides scarce under ambient pressure. Fortunately, high pressure can effectively stimulate the chemical reactivity of elements, making the reaction between hydrogen and late transition metals come true. Previous experimental investigations have ascertained that high pressure can be utilized to synthesize new Pt, Rh and Ir hydrides^12–16^ and the preceding first-principles calculations have predicted the high pressure structures produced through the reaction of the late transition metals (Ru, Rh, Pd, Ag, Os, Ir, Pt, and Au) with hydrogen^17,18^. More interestingly, RhH_2_ is suggested to be a hydrogen storage compound for the highly theoretical volumetric hydrogen density of 163.7 g H_2_/L^15^. On the other hand, as for the early transition metals possessing the smaller electronegativity than hydrogen, they are found to form easily hydrides at ambient conditions, such as ScH_2_, YH_2_/YH_3_, TiH_2_, ZrH_2_, HfH_2_, VH_2_, NbH_2_^19,20^. It is obviously seen that these early transition metal hydrides generally form transition-metal dihydrides TMH_2_. Upon compression, these transition-metal dihydrides that can exist under ambient conditions present fascinating properties that cannot be found at normal conditions. For example, a theoretical study indicated that TiH_2_ at high pressure takes a structural sequence of *I*4/*mmm* $\to$ *P*4/nmm $\to$ *P*2_1_/*m* with the corresponding transformation pressures of 63 and 294 GPa^21^. A subsequent experiment^22^ on ZrH_2_ have offered a new approach with nonhydrostatic compression or shear stress for seeking higher volumetric density hydrogen structures in transition-metal hydrides. Later on, they reported another theoretical investigation on HfH_2_ that it undergoes pressure-induced structural phase transition *I*4/*mmm* $\to$ *Cmma* $\to$ *P*2_1_/*m* at 180 and 250 GPa, respectively, and HfH_2_ is classified as a ionic crystal with the charges transferring from Hf atom to H^23^.

With regard to group 5 element dihydrides, the pressure-induced structural transition and related structural and electronic properties of NbH_2_ and TaH_2_ has also been deeply explored. Gao *et al*. predicted that NbH_2_ transforms from the fluorite *Fm*-3*m* structure into the orthorhombic *Pnma* phase at 50 GPa^24^. Subsequently, we also confirmed the high-pressure phase transition of NbH_2_ and further found a combination of ionic and metallic bonds in these two stable phases (the *Fm*-3*m* and *Pnma* phases)^25^. In the latest theoretical work, TaH_2_ is found to transform from the *P*6_3_*mc* structure into the *Pnma* phase at about 95 GPa^26^. Up to now, despite few theoretical researches on the high-pressure phase transition of VH_2_^27,28^, there is relatively little investigation on its new structures, mechanical properties, hardness and the chemical bonding nature under high pressure. This stimulates us to implement a comprehensive search on the crystal structures for vanadium dihydrides under high pressure and study the related structural, mechanical and electronic properties of newly stable VH_2_ structures.

In the current work, we explore the crystal structures of VH_2_ within the pressure range of 0–300 GPa by employed the developed Crystal Structure Analysis by Particle Swarm Optimization (CALYPSO) method in combination with first-principles calculations. Two stable structures (the *Fm-*3*m* and *Pnma* structures) are confirmed within the stable pressure ranges of 0−46.37 and 46.37−300 GPa, respectively. The *Fm-*3*m* structure is comprised of regular VH_8_ cubes while the *Pnma* structure contains highly distorted VH_9_ tetrakaidecahedrons. The mechanical and dynamical stabilities of both phases are verified with the calculated elastic constants and phonon dispersions, respectively. In addition, the analyses of the Poisson’s ratio, electron localization function (ELF) and Bader charge indicate that both phases are ionic crystals with the charges transferring from V atom to H. Furthermore, the mechanical properties and hardness were also investigated for both phases. The present results can provide a better understanding of the phase transformations, the structural characteristics and bonding character of VH_2_ and promote further experimental and theoretical investigations on the transition metal hydrides.

## Computational method

To search for the ground-state structures for VH_2_ under high pressure, we performed an unbiased structure prediction based on the particle swarm optimization algorithm as implemented with the CALYPSO code^29,30^. The superior efficiency of this methodology has been verified on various systems^25,31–39^. Our structure searches with unit cells containing up to six formula units (f.u.) per simulation cell were conducted within pressures of 0−300 GPa. The detailed description for this search algorithm can be found elsewhere^29,30^. The structural optimization and electronic structure calculations were carried out using density functional theory within the Perdew−Burke−Ernzerhof (PBE) exchange-correlation functional as implemented in the Vienna ab initio simulation package (VASP)^40,41^. Plane-wave basis sets and the projector augmented wave (PAW) method^42^ were adopted with 4*s*^2^3*d*^3^ and 1*s*^1^ treated as valence electron space for V and H atoms, respectively. The cutoff energy of 800 eV for the expansion of the expansion of the wave function into plane waves and appropriate Monkhorst−Pack k-meshes^43^ were selected to ensure that all the enthalpy calculations were well converged to better than 1 meV/atom. The phonon dispersion curves were calculated within the direct supercell approach as implemented in the Phonopy code^44,45^. The electron localization function (ELF)^46,47^ and Bader charge^48^ were also computed using VASP. In addition, all the crystal structures and electron localization functions (ELF) diagrams were generated using VESTA 3.4.4^49^. The elastic constants were determined by calculating stress tensor generated by applying a small strain to an optimized unit cell. Besides, the bulk modulus (*B*), shear modulus (*G*), Young’s modulus (*E*), and Poisson’s ratio were thus derived from the Voigt−Reuss−Hill (VRH) approximation^50^.

## Results and Discussions

### Crystal structure searching and phase transition under pressure

**Figure 1:**
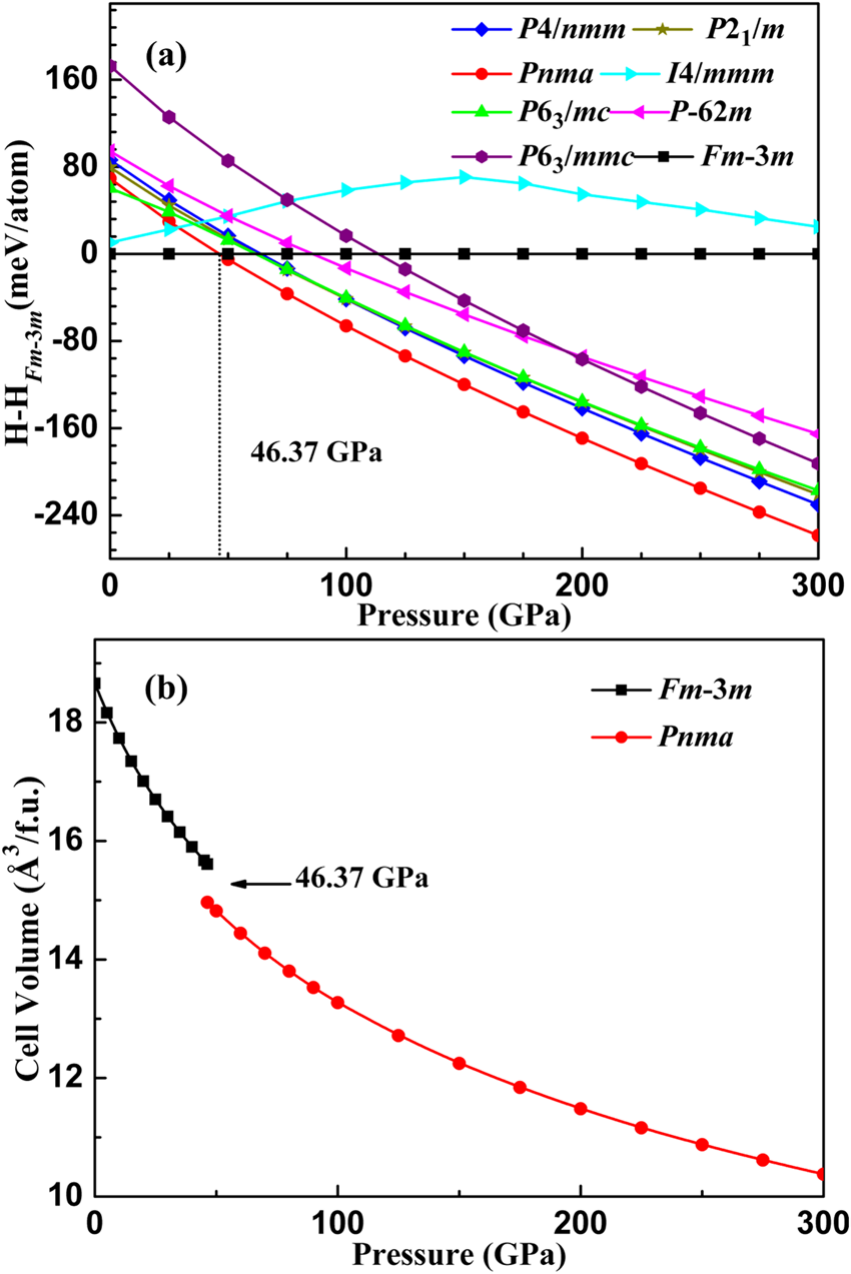
(**a**) Enthalpy-pressure relation with respect to the *Fm-*3*m* phase and (**b**) volume-pressure relations for VH_2_.

Firstly, on the basis of the chemical constitution, we have conducted the structure prediction simulations of VH_2_ in the pressure range of 0−300 GPa. Subsequently, the analysis of the predicted structures provides eight candidate structures with space groups *Fm-*3*m*, *Pnma*, *P*2_1_/*m*, *P*-62*m*, *P*6_3_*mc*, *P*6_3_/*mmc*, *P*4*/nmm* and *I*4*/mmm*. The candidate crystal structures diagrams are depicted in Fig. S1 in the Supplementary Information and their lattice parameters and atomic coordinates under ambient pressure are summarized in Table S1. The fact that all the earlier known structures (*Fm-*3*m*, *Pnma*, *P*6_3_*mc*, *P*6_3_/*mmc*, *I*4*/mmm* and *P*4*/nmm*)^27,28,51–53^ were successfully reproduced in specific pressure ranges supports the validity of CALYPSO methodology used in structure searches of VH_2_. The enthalpy−pressure (H − P) relations of the candidate structures under the pressure range of 0−300 GPa for VH_2_ are presented in Fig. 1a. Under ambient pressure, the cubic CaF_2_ structure (space group *Fm-*3*m*) possesses the lowest ground state energy, which is consistent with the experimental consequence^51^. With increasing pressure, the orthorhombic *Pnma* structure is favored over the *Fm-*3*m* phase above 46.37 GPa, which remains stable in the ground state up to at least 300 GPa. This *Pnma* structure is also the high-pressure configuration of NbH_2_^25,54^, WH_2_^55^ and certain alkaline-earth dihydrides such as CaH_2_, SrH_2_, and BaH_2_^56,57^. This phase transformation is also supported by the volume-pressure relations (see Fig. 1b), as we observed a volume collapse of 4.1% at the transition pressure. Furthermore, the transition pressure (46.37 GPa) is in good agreement with the relevant theoretical calculations^27,28^. Below we concentrate on the relevant properties of the *Fm-*3*m* phase at 0 GPa and the *Pnma* phase at 100 GPa, which are most stable and accessible to experiment at the corresponding pressures.

### Structural properties and the lattice parameter behavior under pressure

**Figure 2:**
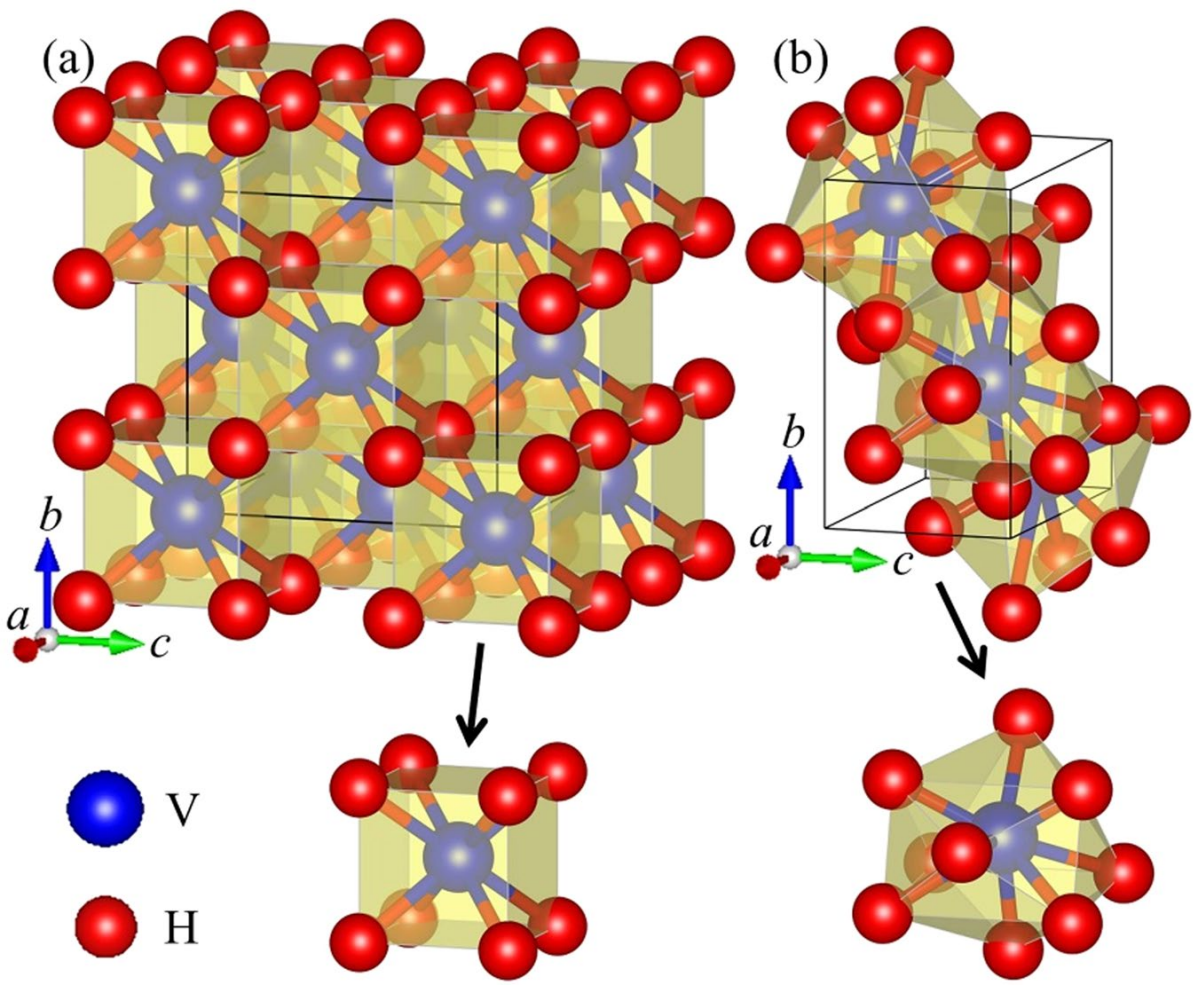
Crystal structures of two considered VH_2_ phases, together with metal coordination polyhedra: (**a**) the *Fm-*3*m* phase at 0 GPa and (**b**) the *Pnma* phase at 100 GPa. The blue and red spheres represent vanadium and hydrogen atoms, respectively, and VH_*n*_ polyhedra are shaded in two phases.

The crystal structures of the *Fm-*3*m* phase at 0 GPa and the *Pnma* phase at 100 GPa for VH_2_ are illustrated in Fig. 2 and their structural parameters are presented in Table 1. For the *Fm-*3*m* phase at 0 GPa, as displayed in Table 1, the lattice parameter (*a* = *b* = *c* = 4.215 Å) is in excellent agreement with the previous results (*a* = *b* = *c* = 4.217 Å) calculated by Chen *et al*.^27^ In the *Fm-*3*m* phase at 0 GPa, each V atom is at the center of a regular VH_8_ cube with eight equal V-H separation of 1.825 Å and identical H-V-H angle of 71°. Therefore, the V environment in the *Fm-*3*m* configuration can be described as highly symmetrical VH_8_ cube. However, in the *Pnma* phase at 100 GPa, each V atom is surrounded by one H atom at a separation of 1.634 Å, two H atoms at a separation of 1.644 Å, one H atom at a separation of 1.654 Å, one H atom at a separation of 1.701 Å, two H atoms at a separation of 1.825 Å and two further H atoms at a separation of 1.899 Å. Besides, the H-V-H angles vary between 46° and 106°. These results describe that the V environment in the *Pnma* phase are strongly distorted VH_9_ tetrakaidecahedron. It is obviously found that the V-H environments in VH_2_ transform from the regular VH_8_ cube to the highly distorted VH_9_ tetrakaidecahedron with increasing pressure. More interestingly, the coordination number of the V atoms in VH_2_ increase monotonously upon further compression. This phenomenon can also be found in other systems^25,31,58^.

**Table 1:** Lattice constants and atomic coordinates of VH_2_ for the *Fm-*3*m* phase at 0 GPa and the *Pnma* phase at 100 GPa.

Pressure (GPa)	Space group	*a*	*b*	*c*	Atomic coordinates (fractional)			
					Atom	*x*	*y*	*z*
0	*Fm-*3*m*	4.215	4.215	4.215	V1(4a)	0.00000	0.00000	0.00000
					H1(8c)	0.25000	0.25000	0.25000
0	*Fm-*3*m*	4.217a	4.217a	4.217a	V1(4a)	0.00000	0.00000	0.00000
					H1(8c)	0.25000	0.25000	0.25000
100	*Pnma*	4.268	2.622	4.721	V1(4c)	0.23761	0.75000	0.09160
					H1(4c)	0.52689	0.75000	0.71430
					H2(4c)	0.12491	0.75000	0.42227

^a^ref. ^27^.

**Figure 3:**
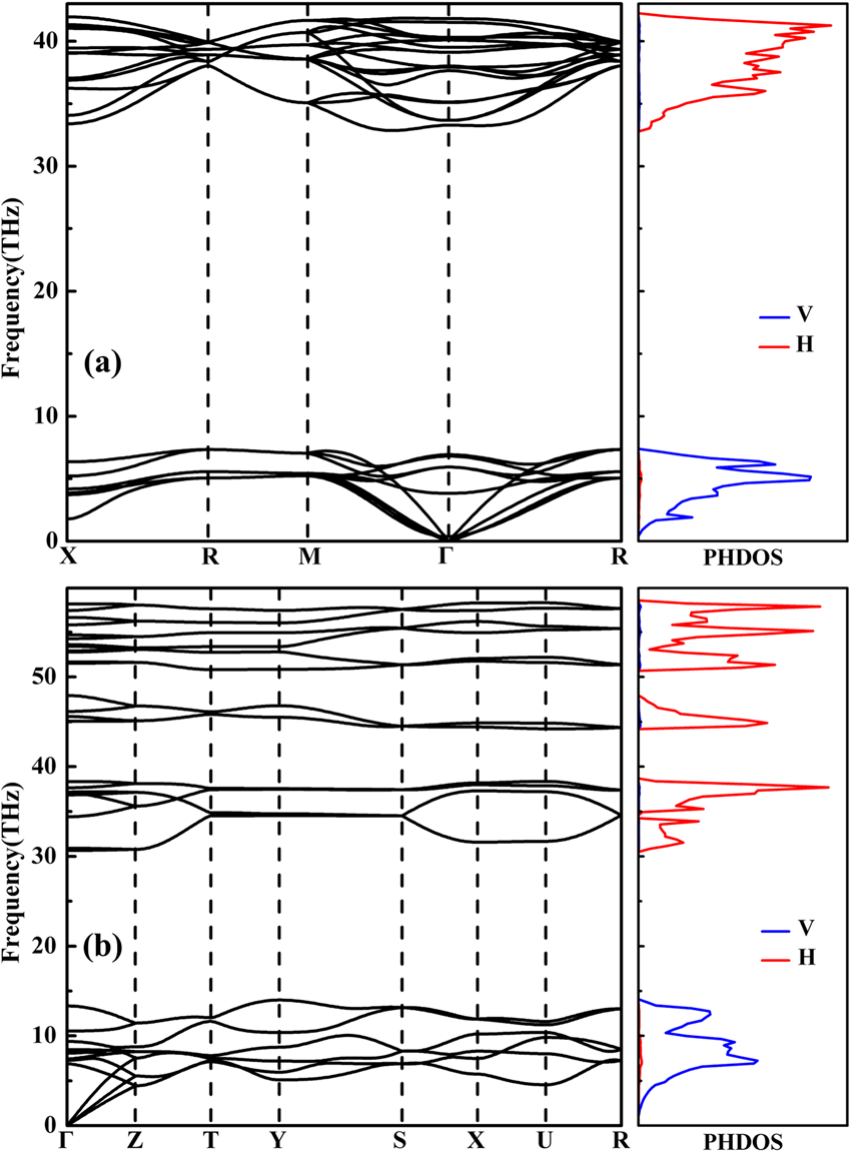
Phonon dispersion curves and projected phonon density of states (PHDOS) for two stable VH_2_ phases: (**a**) the *Fm-*3*m* phase at 0 GPa and (**b**) the *Pnma* phase at 100 GPa.

It is essential to calculate the phonon spectra of two stable VH_2_ structures for the purpose of ascertaining their dynamical stabilities. The calculated phonon dispersion curves and projected phonon density of states (PHDOS) for the *Fm*-3*m* phase at 0 GPa and the *Pnma* phase at 100 GPa are depicted in Fig. 3. The absence of any imaginary frequencies in the entire Brillouin zone confirms the dynamical stabilities of both structures. Notably, the phonon bands can be divided into two separate regions. The higher frequency modes are mainly correspond to the vibration of the H atoms while the lower frequency region is mostly related to the V atoms, which can be attributed to the much heavier atom mass of vanadium atom than hydrogen atom.

For the purpose to investigate the effect of the external pressure on the lattice parameters and the volume of VH_2_, the normalized parameters *a*/*a*_0_, *b*/*b*_0_, *c*/*c*_0_ and volume *V*/*V*_0_ of two considered phases as a function of pressure are displayed in Fig. S2, where *a*_0_, *b*_0_, *c*_0_ and *V*_0_ are the equilibrium structural parameters at zero pressure, respectively. It is obviously found that the values *a*/*a*_0_, *b*/*b*_0_, *c*/*c*_0_ and *V*/*V*_0_ decrease gradually with the increasing pressure. This can be attributed to the reason that the distance between atoms is reduced as the pressure increases, which make the repulsive interaction among atoms strengthened and lead to the higher incompressibility of the crystal under pressure.

### Mechanical stability, mechanical properties and hardness

To inspect the mechanical stabilities of two stable VH_2_ structures, we computed the elastic constants of the *Fm-*3*m* phase at 0 GPa and the *Pnma* phase at 100 GPa by using the strain−stress means^59^, as summarized in Table S2. As given in Table S2, both phases satisfy their respectively mechanical stability standards^60^, which states that both structures are mechanically stable.

In addition, to get further knowledge of mechanical properties for two stable configurations, the elastic constants of the *Fm-*3*m* and *Pnma* structures of VH_2_ at 0 GPa were calculated with the identical strain−stress means^59^. At the same time, the bulk modulus (*B*) and shear modulus (*G*), *B/G*, Young′s modulus *E* (GPa) and Poisson’s ratio (*ν*) can be derived from the calculated elastic constants on the basis of the Voigt–Reuss–Hill method^61^. The computed consequences above are tabulated in Table 2. Interestingly, the value of *C*_11_ for two considered crystals is larger than the others. It is well known that a material with a high bulk modulus indicates its strong ability to resist volume deformation caused by an applied load. According to Table 2, the calculated bulk moduli of the *Fm-*3*m* and *Pnma* structures are 174 and 173 GPa, respectively, illustrating the incompressible feature of these two phases to a certain degree. In addition, the shear modulus of a material can be used to quantify its resistance to the shear deformation. From Table 2, these two crystals possess the equal the shear modulus of 118 GPa, suggesting that both configurations can withstand the shear strain to a large extent. The ratio value of *B*/*G* is commonly used to describe the ductility or brittleness of materials with 1.75 as the critical value^60^. From Table 2, the calculated identical ratio value of *B*/*G* for both phases is 1.47, indicating their brittle characteristic. Poisson’s ratio is an important parameter to describe the bond nature in a material. For covalent materials, the value of *ν* should be small (typically *ν* = 0.1), and shear modulus *G* should be slightly different with bulk modulus *B* with *G* = 1.1*B*. The typical value of *ν* for ionic materials is 0.25 and *G* = 0.6*B*. For metallic materials, *ν* is typically 0.33 and *G* = 0.4*B*; in the extreme case where *ν* is 0.5, G is zero^62^. From Table 2, the equal *ν* (0.22) for both structures extremely approach the typical value *ν* (0.25) of ionic materials. Further, shear modulus *G* is equal to 0.68*B*. These two conditions above demonstrate that the *Fm-*3*m* and *Pnma* phases of VH_2_ can be classified into ionic materials. In order to estimate the hardness (*Hv*) of VH_2_, an empirical hardness equation is applied as follows: *H*_*v*_ = 2(*K*^2^*G*)^0.585^ − 3, where *K* = *G*/*B*^63^. As shown in Table 2, the calculated hardness (*H*_*v*_) values of the *Fm-*3*m* and *Pnma* phases are 17.83 and 17.68 GPa, respectively, declaring that both phases of VH_2_ could be classified as incompressible and hard materials.

**Table 2:** Calculated elastic constants *C*_*ij*_ (GPa), bulk modulus *B* (GPa), shear modulus *G* (GPa), *B/G*, Young’s modulus *E* (GPa), Poisson’s ratio *ν* and Vickers hardness H_*v*_ of VH_2_ for the *Fm-*3*m* and *Pnma* phases under 0 GPa.

Phase	C_11_	C_22_	C_33_	C_44_	C_55_	C_66_	C_12_	C_13_	C_23_	B	G	B/G	E	ν	Hv
Fm-3m	293			143			115			174	118	1.47	290	0.22	17.83
Pnma	340	332	337	106	121	119	101	81	93	173	118	1.47	288	0.22	17.68

### Electronic properties and bonding features

**Figure 4:**
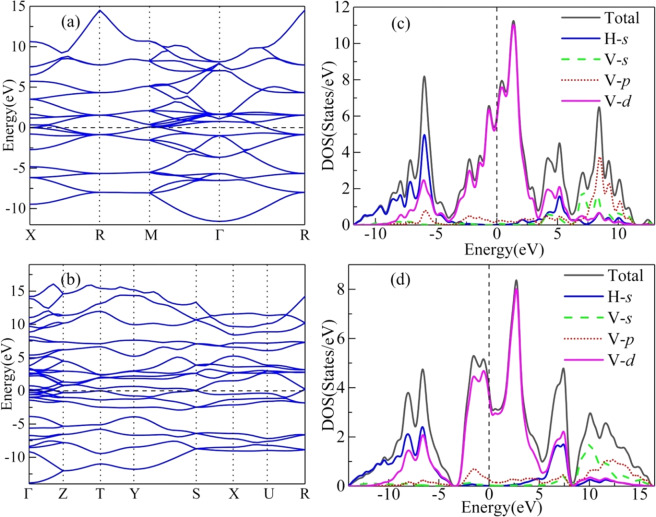
Calculated band structures and densities of states (DOS) for two stable VH_2_ phases. The electronic band structures for (**a**) the *Fm-*3*m* phase at 0 GPa and (**b**) the *Pnma* phase at 100 GPa. The total and partial density of states for (**c**) the *Fm-*3*m* phase at 0 GPa and (**d**) the *Pnma* phase at 100 GPa.

In order to further gain insight into the electronic properties of both stable VH_2_ configurations, we calculated their electronic band structures and densities of states (DOS) (see Fig. 4). Both structures are found to exhibit metallic character to the overlap between the conduction bands and the valence bands, as displayed in Fig. 4a,b. This is also confirmed by the finite electronic DOS at the Fermi level (*E*_*F*_) in Fig. 4c,d. For both compounds, the total densities of states (TDOS) near the Fermi level is largely contributed by the V-*d* states, so the metallic properties are mainly due to partially filled V 3*d* shell.

**Figure 5:**
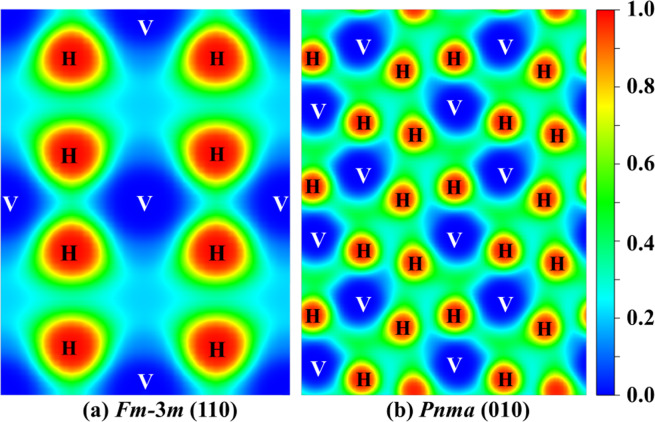
Electron localization function (ELF) of two stable VH_2_ phases: (**a**) (110) plane for the *Fm-*3*m* phase at 0 GPa and (**b**) (010) plane for the *Pnma* phase at 100 GPa.

To further visualize the chemical bonding nature in the polymeric sublattice for two considered VH_2_ structures, we calculate the electron localization function (ELF) which can make a description of the bond type between atoms, as shown in Fig. 5. Generally speaking, large ELF values (>0.5) correspond to covalent bonds and lone pair or inner shell electrons while smaller ELF values (<0.5) correspond to ionic or metallic bonds^46,47^. For two stable VH_2_ structures, the analyses of ELF toward the neighboring V − H and H − H connections obviously reveal no electron localization, thus suggesting that no covalent interaction exists for neighboring V − H and H − H. Moreover, the ELF values between V and the nearest H atom in both stable phases are smaller than 0.5, implying the ionic or metallic bonds are present between V and H atoms. Furthermore, in order to illuminate the chemical bonding features between V and H atoms, Bader charge analysis^48^ was implemented, as summarized in Table 3. For the *Fm-*3*m* phase at 0 GPa, each H atom gains 0.54 *e* while the V atoms lose 1.08 *e*. In addition, for the *Pnma* phase at 100 GPa, a charge of 0.92 *e* is stripped off the V atom and transferred completely to the H atoms (0.46 *e* each). The current prediction of the charge transferring from V to H atoms can also be corroborated by the element electronegativity. From the periodic law^64^, the electronegativity value of the V element (1.63) is smaller than that of the H element (2.2), meaning that the charge should transfer from V to H atoms. The calculated consequences above apparently show that there are large amounts of charge transferring from V to H atoms, demonstrating the ionic character of the V−H bonding in two stable VH_2_ crystals. This ionic bonding character is similar in AlH_3_, LiH_*n*_, HfH_2_, TaH_*n*_, VH, VH_3_ and VH_5_^23,26,65–67^. However, the ionic characteristic for the H atoms is different from that of many hydrogen-rich polymorphs (i.e., SiH_4_, GeH_4_, SnH_4_, InH_3_, OsH_6_ and OsH_8_)^68–72^, where the H atoms are either bonded to the nearest H atoms to form an H_2_ or H_3_ unit and/or they are covalently bonded to M (M = Si, Ge, Sn, In, or Os) atoms to form M − H bonds. In short, a combination analysis of ELF and Bader charge reveals that the ionic bonds are formed between V and H atoms and VH_2_ should be classified as an ionic crystal, which is accord with the analysis of Poisson’s ratio above.

**Table 3:** Calculated Bader charges of V and H atoms in two stable VH_2_ crystals.

Phase	Pressure(GPa)	Atom	Charge value (e)	*δ*(*e*)
*Fm-*3*m*	0	V	3.92	1.08
		H	1.54	−0.54
*Pnma*	100	V	4.08	0.92
		H	1.46	−0.46

δ represents the amount of charge transferred from V atom to H atom.

## Conclusions

In summary, we conducted the systematic structure evolutionary searches of the VH_2_ compound based on the CALYPSO method for crystal structure prediction combined with first-principles calculations under the pressure range between 0 and 300 GPa. This material undergoes a pressure-induced structural phase transformation from the cubic structure *Fm-*3*m* structure to the orthorhombic *Pnma* structure at 46.37 GPa. Intriguing, the *Fm-*3*m* structure is comprised of regular VH_8_ cubes while the *Pnma* structure contains highly distorted VH_9_ tetrakaidecahedron. The calculations of elastic constants and phonon dispersions confirm the mechanical and dynamical stabilities of both configurations, respectively. Both phases possess the metallic properties as verified by the band structures and densities of states for two stable VH_2_ structures. Further analyses of the Poisson’s ratio, ELF and Bader charge indicate that VH_2_ should be classified into an ionic crystal with large amounts of charge transferring from V atom to H. Remarkably, the estimated hardness values of the *Fm*-3*m* and *Pnma* phases are 17.83 and 17.68 GPa, respectively. The current work will stimulate further high pressure experiments to synthesize these vanadium hydrides and carry out the structural, mechanical and hardness measurements.

## Supplementary information

Supplementary information.

## References

[1] Eremets MI (2008). Superconductivity in hydrogen dominant materials: silane. Science.

[2] Pickard CJ, Needs RJ (2006). High-pressure phases of silane. Phys. Rev. Lett..

[3] Jin X (2010). Superconducting high-pressure phases of disilane. Proc. Natl. Acad. Sci. U. S. A..

[4] Gao G (2008). Superconducting high pressure phase of germane. Phys. Rev. Lett..

[5] Tse J, Yao Y, Tanaka K (2007). Novel Superconductivity in Metallic SnH_4_ under high pressure. Phys. Rev. Lett..

[6] Duan D (2014). Pressure-induced metallization of dense (H_2_S)_2_H_2_ with high-T_*c*_ superconductivity. Sci. Rep.

[7] Drozdov P (2015). Conventional superconductivity at 203 kelvin at high pressures in the sulfur hydride system. Nature.

[8] Einaga M (2016). Crystal structure of the superconducting phase of sulfur hydride. Nat. Phys.

[9] Mao WL, Mao HK (2004). Hydrogen storage in molecular compounds. Proc. Natl. Acad. Sci. U. S. A..

[10] Ying J (2019). Synthesis and stability of tantalum hydride at high pressures. Phys. Rev. B..

[11] Li H (2019). Superconducting TaH_5_ at high pressure. New J. Phys.

[12] Degtyareva O (2009). Formation of transition metal hydrides at high pressures. Solid State Commun.

[13] Scheler T (2011). Synthesis and properties of platinum hydride. Phys. Rev. B.

[14] Antonov V (2002). Phase transformations, crystal and magnetic structures of high-pressure hydrides of *d*-metals. J.Alloys Compd..

[15] Li B (2011). Rhodium dihydride (RhH_2_) with high volumetric hydrogen density. Proc. Natl. Acad. Sci. U. S. A..

[16] Scheler T (2013). High-pressure synthesis and characterization of iridium trihydride. Phys. Rev. Lett..

[17] Zaleski-Ejgierd P (2014). Formation of transition metal hydrides at high pressures. Phys. Chem. Chem. Phys..

[18] Gao G (2012). Pressure-induced formation of noble metal hydrides. J. Phys. Chem. C.

[19] Smithson H (2002). First-principles study of the stability and electronic structure of metal hydrides. Phys. Rev. B.

[20] Miwa K, Fukumoto A (2002). First-principles study on 3*d* transition-metal dihydrides. Phys. Rev. B.

[21] Gao G (2013). Pressure induced phase transitions in TiH_2_. J. Appl. Phys..

[22] Huang X (2014). Structural stability and compressive behavior of ZrH_2_ under hydrostatic pressure and nonhydrostatic pressure. RSC Adv.

[23] Liu Y (2015). First-principles study on the structural and electronic properties of metallic HfH_2_ under pressure. Sci. Rep.

[24] Gao G (2013). Theoretical study of the ground-state structures and properties of niobium hydrides under pressure. Phys. Rev. B.

[25] Zhang C (2017). Prediction of novel high-pressure structures of magnesium niobium dihydride. ACS Appl. Mater. Interfaces.

[26] Zhuang Q (2017). Pressure-stabilized superconductive ionic tantalum hydrides. Inorg. Chem..

[27] Chen C (2014). Pressure induced phase transition in MH_2_ (M = V, Nb),. J. Chem. Phys..

[28] Li X, Peng F (2017). Superconductivity of pressure-stabilized vanadium hydrides. Inorg. Chem..

[29] Wang Y, Lv J, Zhu L, Ma Y (2010). Crystal structure prediction via particle-swarm optimization. Phys. Rev. B: Condens. Matter Mater. Phys.

[30] Wang Y, Lv J, Zhu L, Ma Y (2012). CALYPSO: A method for crystal structure prediction. Comput. Phys. Commun..

[31] Lu C, Miao MS, Ma YM (2013). Structural evolution of carbon dioxide under high pressure. J. Am. Chem. Soc..

[32] Zhu L (2014). Reactions of xenon with iron and nickel are predicted in the earth’s inner core. Nat. Chem.

[33] Lu SH (2014). Self-assembled ultrathin nanotubes on diamond (100) surface. Nat. Commun..

[34] Gao B (2019). Interface structure prediction via CALYPSO method. Sci. Bull.

[35] Zhang CZ (2015). Prediction of stable ruthenium silicides from first-principles calculations: stoichiometries, crystal structures, and physical properties. ACS Appl. Mater. Interfaces.

[36] Lu C (2017). Extraordinary indentation strain stiffening produces superhard tungsten nitrides. Phys. Rev. Lett..

[37] Lu C, Chen CF (2018). High-pressure evolution of crystal bonding structures and properties of FeOOH. J. Phys. Chem. Lett..

[38] Lu C, Amsler M, Chen CF (2018). Unraveling the structure and bonding evolution of the newly discovered iron oxide FeO_2_. Phys. Rev. B.

[39] Tang X (2019). CoB_6_ monolayer: A robust two-dimensional ferromagnet. Phys. Rev. B.

[40] Kresse G, Furthmüller J (1996). Efficient iterative schemes for *ab initio* total-energy calculations using a plane-wave basis set. Phys. Rev. B: Condens. Matter Mater. Phys..

[41] Perdew JP, Burke K, Ernzerhof M (1996). Generalized gradient approximation made simple. Phys. Rev. Lett..

[42] Kresse G, Joubert D (1999). From ultrasoft pseudopotentials to the projector augmented-wave method. Phys. Rev. B: Condens. Matter Mater. Phys..

[43] Monkhorst HJ, Pack JD (1976). Special points for brillouin-zone integrations. Phys. Rev. B.

[44] Parlinski K, Li ZQ, Kawazoe Y (1997). First-principles determination of the soft mode in cubic ZrO_2_. Phys. Rev. Lett..

[45] Togo A, Oba F, Tanaka I (2008). First-principles calculations of the ferroelastic transition between rutile-type and CaCl_2_-type SiO_2_ at high pressures. Phys. Rev. B.

[46] Becke AD, Edgecombe KE (1990). A simple measure of electron localization in atomic and molecular systems. J. Chem. Phys..

[47] Savin A (1992). Electron localization in solid-state structures of the elements: the diamond structure. Angew. Chem., Int. Ed. Engl.

[48] Bader, R. F. Atoms in Molecules: A Quantum Theory (1994).

[49] Momma, K. & Izumi, F. VESTA 3 for Three-dimensional visualization of crystal, volumetric and morphology data. *J. Appl. Crystallogr*. **44**, 1272–1276, http://jp-minerals.org/vesta/en/ (2011).

[50] Hill R (1952). The elastic behavior of a crystalline aggregate. Proc. Phys. Soc. London, Sect. A.

[51] Reilly JJ, Wiswall RH (1970). The higher hydrides of vanadium and niobium. Inorg. Chem..

[52] Smithson H (2002). First-principles study of the stability and electronic structure of metal hydrides. Phys. Rev. B..

[53] Miwa K (2002). First-principles study on 3*d* transition-metal dihydrides. Phys. Rev. B..

[54] Gao G (2013). Theoretical study of the ground-state structures and properties of niobium hydrides under pressure. Phys. Rev. B: Condens. Matter Mater. Phys.

[55] Scheler T (2013). Nanocrystalline tungsten hydrides at high pressures. Phys. Rev. B: Condens. Matter Mater. Phys.

[56] Li Y (2008). High-pressure phase transformations in CaH2. J. Phys.: Condens. Matter.

[57] El Gridani AH (2002). Elastic, electronic and crystal structure of BaH_2_: A pseudopotential study. J. Mol. Struct.: Theochem.

[58] Zhong X (2015). Ten-fold coordinated polymorph and metallization of TiO_2_ under high pressure. RSC Adv.

[59] Lu C (2010). Theoretical investigation on the high-pressure structural transition and thermodynamic properties of cadmium oxide. Europhys. Lett..

[60] Wu Z (2007). Crystal structures and elastic properties of superhard IrN_2_ and IrN_3_ from first principles. Phys. Rev. B: Condens. Matter Mater. Phys.

[61] Connétable D, Thomas O (2009). First-principles study of the structural, electronic, vibrational, and elastic properties of orthorhombic NiSi. Phys. Rev. B.

[62] Haines J, Léger JM, Bocquillon G (2001). Synthesis and design of superhard materials. Annu. Rev. Mater. Res..

[63] Chen XQ (2011). Modeling hardness of polycrystalline materials and bulk metallic glasses. Intermetallics.

[64] Lide, D. R. Handbook of Chemistry and Physics, 82th Edition, CRC Press, Boca Raton, FL, (2002).

[65] Pickard J, Needs RJ (2007). Metallization of aluminum hydride at high pressures: A first-principles study. Phys. Rev. B: Condens. Matter Mater. Phys.

[66] Zurek E (2009). A little bit of lithium does a lot for hydrogen. Proc. Natl. Acad. Sci. U. S. A..

[67] Zhuang Q (2017). Investigation of superconductivity in compressed vanadium hydrides. Phys. Chem. Chem. Phys..

[68] Zhang H (2015). High-temperature superconductivity in compressed solid silane. Sci. Rep.

[69] Zhang H (2015). Investigation of stable germane structures under high-pressure. Phys. Chem. Chem. Phys..

[70] Zhang H (2016). Pressure-induced phase transition of SnH_4_: a new layered structure. RSC Adv.

[71] Liu Y (2015). Pressure-induced structures and properties in indium hydrides. Inorg. Chem..

[72] Liu Y (2015). Structures and properties of osmium hydrides under pressure from first principle calculation. J. Phys. Chem. C.

