# Supplementary material for: Structural, mechanical and electronic properties and hardness of ionic vanadium dihydrides under pressure from first-principles computations
Source: Sci Rep. 2020 Jun 1;10:8868. doi: 10.1038/s41598-020-65910-4 (PMC7264295; doi:10.1038/s41598-020-65910-4)
Supplement: Supplementary file 1 — Supplementary information. [file 41598_2020_65910_MOESM1_ESM.pdf]

Supplementary Information

Structural, mechanical and electronic properties and hardness of ionic vanadium dihydrides under pressure from first-principles computations

Wenjie Wang[1], Chuanzhao Zhang[1*], Yuanyuan Jin[1*], Song Li[1], Weibin Zhang[1], Panlong Kong[2], Chengwu Xie[1], Chengzhuo Du[1], Qian Liu[1] & Caihong Zhang[1]

[1]Department of Physics and Optoelectronic Engineering, Yangtze University, Jingzhou 434023, China.

[2]School of Physical Science and Technology, Key Laboratory of Advanced Technologies of Materials, Southwest Jiaotong University, Chengdu 610031, China

Correspondence and requests for materials should be addressed to C.Z.Z (email: zcz19870517@163.com) or Y.Y.J. (email: scujyy@163.com)

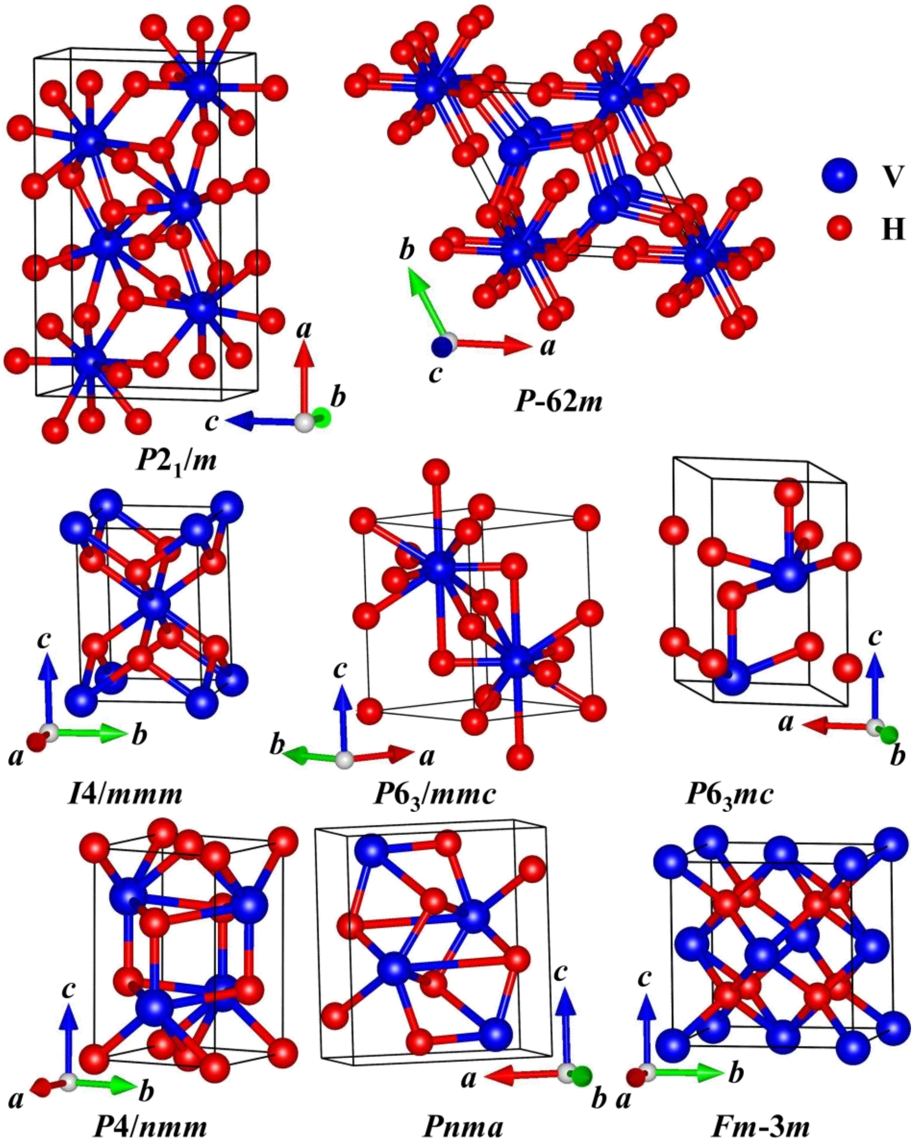

Figure S1. The predicted phases of VH_2. In all figures the V atoms are blue and the H atoms are red.

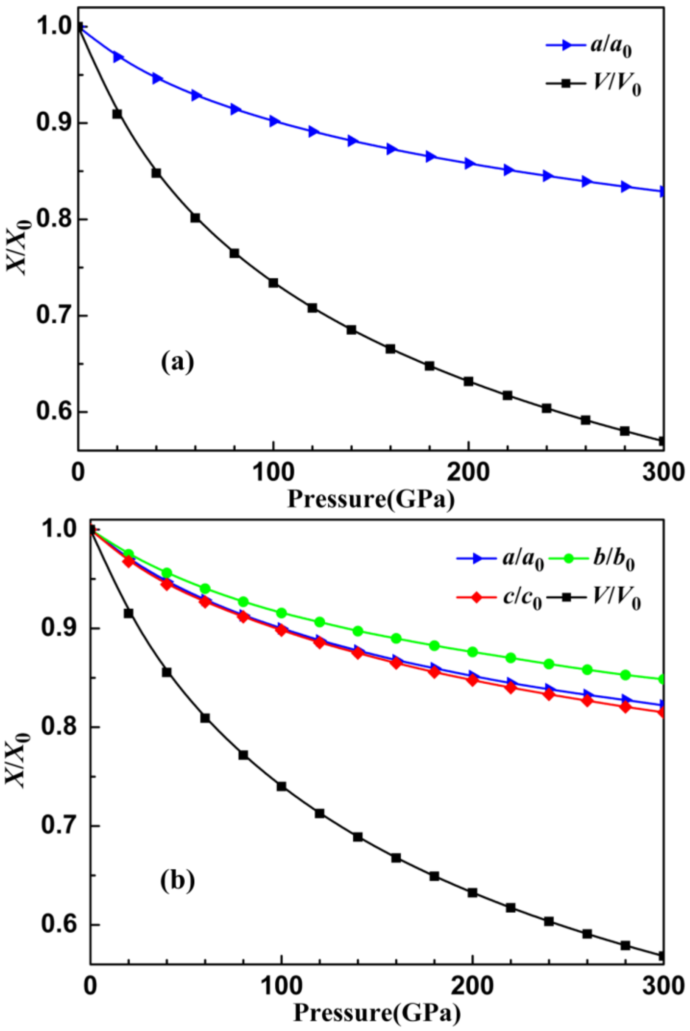

Figure S2. The normalized parameters X/X_0(X =a, b, c and V) of VH_2 for (a) the *Fm*-3*m* phase and (b) the *Pnma* phase as a function of pressure.

Table S1. Structural information of the predicted structures for VH_2 under ambient pressure.

Space group	Lattice parameters (Å, °)	Atomic coordinates (fractional)			
		Atom	*x*	*y*	*z*
Fm-3*m*	*a*=*b*=*c*=4.215	V1(4a)	0.00000	0.00000	0.00000
		H1(8c)	0.25000	0.25000	0.25000
Pnma	*a*=4.742, *b*=2.856, *c*=5.288	V1(4c)	0.24111	0.75000	0.09654
		H1(4c)	0.53394	0.75000	0.69001
		H2(4c)	0.12421	0.75000	0.42049
$P2_1/m$	*a*=8.047, *b*=2.887, *c*=4.624 β=89.659	V1(2e)	0.90308	0.75000	0.24389
		V2(2e)	0.43136	0.75000	0.76292
		V3(2e)	0.75179	0.25000	0.74820
		H1(2e)	0.45784	0.75000	0.37435
		H2(2e)	0.90650	0.75000	0.84887
		H3(2e)	0.92353	0.25000	0.49824
		H4(2e)	0.78408	0.25000	0.12710
		H5(2e)	0.37040	0.25000	0.04842
		H6(2e)	0.28683	0.25000	0.53468
P-62*m*	*a*=*b*=4.676, *c*=2.845, γ=120	V1(2d)	0.33333	0.66667	0.50000
		V2(1a)	0.00000	0.00000	0.00000
		H1(3g)	0.25190	0.00000	0.50000
		H2(3f)	0.40197	0.40197	0.00000
$P6_3mc$	*a*=*b*=2.967, *c*=4.838, γ=120	V1(2b)	0.33333	0.66667	0.55255
		H1(2a)	0.00000	0.00000	0.17165
		H2(2b)	0.66667	0.33333	0.41920
$P6_3/mmc$	*a*=*b*=3.011, *c*=4.494, γ=120	V1(2d)	0.33333	0.66667	0.75000
		H1(2c)	0.33333	0.66667	0.25000
		H2(2a)	0.00000	0.00000	0.00000
P4/nmm	*a*=*b*=2.860, *c*=4.397	V1(2b)	0.00000	0.50000	0.26513
		H1(2a)	0.00000	0.50000	0.66559
		H2(2b)	0.00000	0.00000	0.00000
I4/mmm	*a*=*b*=2.982, *c*=4.211	V1(2a)	0.00000	0.00000	0.00000
		H1(4d)	0.00000	0.50000	0.25000

Table S2. Calculated elastic constants C_{ij} (GPa) for two stable VH_2 phases: the *Fm*-3*m* phase at 0 GPa and the *Pnma* phase at 100 GPa.

Pressure (GPa)	Phase	C_{11}	C_{22}	C_{33}	C_{44}	C_{55}	C_{66}	C_{12}	C_{13}	C_{23}
0	*Fm*-3*m*	293			143			115		
100	*Pnma*	740	729	779	170	190	255	341	331	351
